# Atypical Afta Major Healing after Photodynamic Therapy

**DOI:** 10.1155/2017/8517470

**Published:** 2017-09-20

**Authors:** Cinzia Casu, Carla Mannu

**Affiliations:** ^1^Private Practice, Cagliari, Italy; ^2^Diabetology, San Michele Hospital, Cagliari, Italy

## Abstract

The aim of this study is to report a case of atypical Afta Major healing in a patient with recurrent aphthous stomatitis (SAR) with a type of photodynamic therapy. A female patient with SAR affected for about 2 years reported a history of hypothyroidism treated with Levothyroxine. The oral cavity clinical examination showed several major symptomatic ulcers, previously treated with topical and systemic therapies without any benefit. The largest of them is present for more than 40 days, in spite of topical cortisone applications, with significant pain symptoms reported by the patient. It was decided to perform a session of photodynamic therapy with a device that emits a LED light used in combination with a photosensitive reagent (Toluidine blue). The dye was applied on the entire surface of the lesion beyond the margins and even encroaching on healthy tissue. The light diode was turned on with a wavelength of 630 nm with cycles from 30 seconds, 10 consecutive times above it. After a few days, a curious phenomenon happened: healing of Afta Major starting from the center, which was almost completely healed towards the borders of the lesion. No previous literature reports this type of healing. Photodynamic therapy could be a successful treatment for SAR.

## 1. Introduction

Recurrent aphthous stomatitis (RAS), also known as a recurrent aphthous ulcer or recurrent oral ulcer, is the most common recurrent oral mucosal lesion. The prevalence of RAS in the general population is between 2% and 50%; most estimates fall between 5% and 25%. RAS clinically manifests as small, round or ovoid, painful, self-healing, and recurrent ulcers with circumscribed margins, erythematous haloes, and yellow or gray floors. RAS may occur during childhood or adolescence, and mucosal lesions may disturb patients' daily activities, such as drinking, eating, and speaking [1]. There are three clinical presentations of RAS: minor RAS, major RAS, and herpetiform ulceration. Minor RAS: it is the most common form of RAS and approximately 85% of patients have lesions of this type. The ulcers are superficial, usually <1 cm in diameter; their size is approximately 4-5 mm in diameter. The classification of minor RAS does not depend on the dimensions of the lesions alone but on a number of other clinical features such number of ulcers from 1 to 5. Major RAS is less common than minor RAS lesions (approximately 10–15% of all RAS). These lesions are similar in appearance to those of minor RAS; however, they are larger than 10 mm in diameter, are deeper, often scarred, and can heal after several weeks. Herpetiform ulceration constitutes only 5–10% of all RAS cases [2]. A resemblance between this term with herpes simplex virus infection exists. Herpetiform ulcers are small (1-2 mm) and multiple ulcers (5–100) may be present at the same time [2]. The etiology of RAS is still unknown. The potential trigger factors include genetic predisposition, viral and bacterial infections, food allergies, vitamin and microelement deficiencies, systemic diseases (e.g., celiac disease, Crohn's disease, ulcerative colitis, and AIDS), increased oxidative stress, hormonal defects, mechanical injuries, and anxiety [3]. There is no agreement in the treatment of RAS; therefore, many therapies have been tried; few have been subjected to double-blind randomized controlled studies. The aim of the treatment of RAS is to decrease symptoms, reduce ulcer number and size, and increase disease-free periods [2]. Conventional treatment for RAS involves the use of topical or systemic drugs. Topical agents, such as corticosteroids and other anti-inflammatory agents, including benzydamine, amlexanox, aphthae, and triclosan, are usually provided for patients with mild symptoms in forms of mouth rinse, adhesive paste, or anesthetic gel. However, for those patients with particularly frequent or severe RAS, systemic immunosuppressive treatment (corticosteroids, pentoxifylline, thalidomide, etc.) may be necessary [3]. Corticosteroids, antibiotics, and analgesics play the role of mainstay in the treatment of patients with RAS, especially in improving healing of severe RAS [1–3]. However, long-term or repeated use of these medications should be avoided as fungal infection or drug resistance or even life-threatening complications may be caused [3]. Therefore, many doctors are exploring new treatments for RAS. Several studies aimed to evaluate the efficacy and safety of topical treatment with natural herbal medicines on recurrent aphthous stomatitis; however, the evidence remains insufficient [4, 5]. Currently, clinical case reports and randomized controlled clinical trials about several different types of lasers (Nd:YAG laser, Er:YAG laser, InGaAlP laser, GaAlAs laser, etc.) are reported for treatment of RAS [3]. No studies on photodynamic therapy are proposed in the literature for the treatment of RAS. The aim of this work is to report an atypical healing of an Afta Major of a patient with RAS with a particular type of photodynamic therapy.

## 2. Materials and Methods

**Figure 1:**
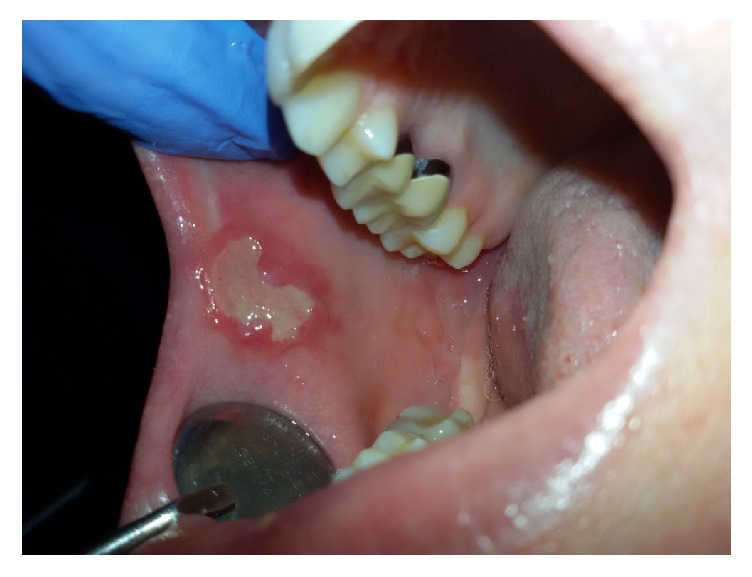
The first presentation of the lesion, after topical corticosteroids applications.

The patient is a 34-year-old Caucasian woman, who presented in September 2014 with a previous diagnosis of recurrent aphthous ulceration from about 2 years. The patient reported a positive medical history of hypothyroidism treated with Levothyroxine 125 mcg (1/day). Examinations showed altered blood chemistry values of PCR (about 4-5 times higher than the threshold parameter) and of serum proteins. Clinical examination of the oral cavity has revealed the presence of several major symptomatic ulcers. Also, she reported having been subjected without any benefit to topical therapies (locorten gel application) and to systemic drugs (deltacortene cpr 25 mg/1/day). In particular an aphthous lesion on the buccal mucosa was present for more than 40 days, in spite of topical cortisone applications, with significant pain symptoms and with repercussions on nutrition reported by the patient (Figure 1). It was decided to perform a session of photodynamic therapy with the application of a light diode and a dye-based Toluidine blue (FotoSan 630). FotoSan 630 (CMS dental, Denmark, Dentalica) is a device that emits a LED light used in combination with a photosensitive reagent (Toluidine blue in syringes with a concentration of 0.1 mg/ml). The basic principle of this therapy is represented by the photochemical reaction between a photosensitive substance (in this case Toluidine blue) and a light source that emits a specific light spectrum (630 nm). The light-sensitive substances applied have very mild affinity with mammalian cells. For this there are no adverse effects during the treatments. The intensity of the light that emitted diodes is between 2000 and 4000 MW/cm2. This device works with three different modalities that correspond to different time cycles of application: 10, 20, and 30 seconds, respectively.

**Figure 2:**
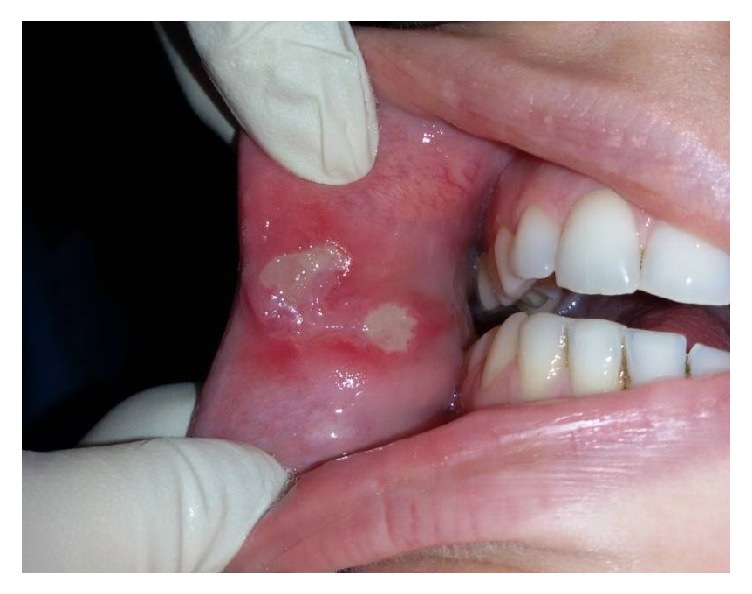
The lesion after light diode applications (wavelength of 630 nm with cycles from 30 seconds, 10 consecutive times).

**Figure 3:**
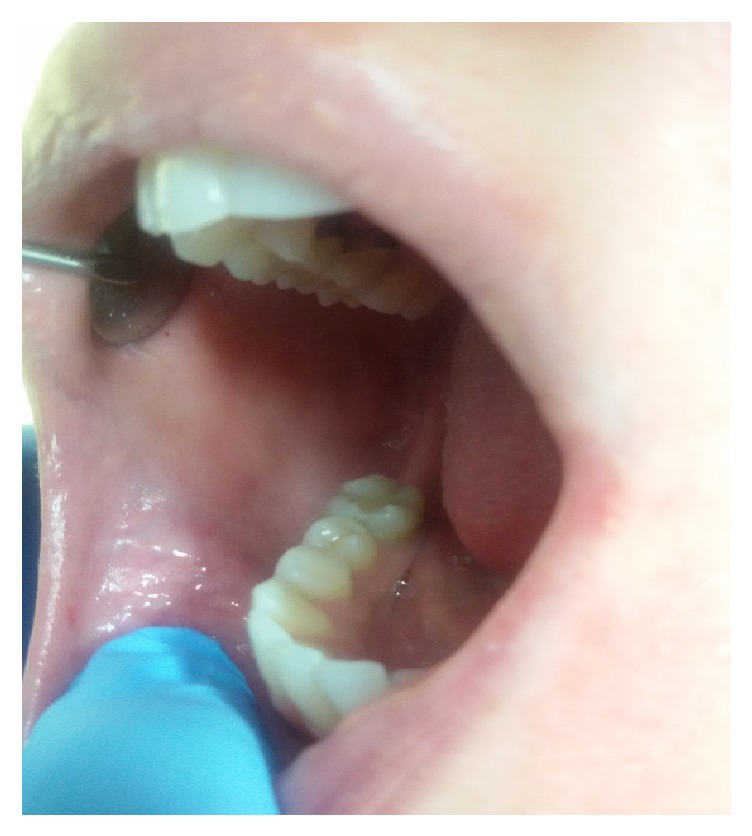
The lesion completely healed after a week.

The dye was applied on the entire surface of the lesion beyond the margins and even encroaching on healthy tissue. The light diode was then turned on with a wavelength of 630 nm with cycles from 30 seconds, 10 consecutive times, using a long pipped tip and performing circular movements of about 0.5 cm above it. At the end of the 10 applications, the dye was completely removed with a gauze and the patient performed a final rinsing with water. After a few days, a curious phenomenon happened: healing of Afta Major starting from the center, which was almost completely healed towards the borders of the lesion (Figure 2). The patient reported some relief a few hours after the appointment. After a week the wound had healed completely (Figure 3).

## 3. Discussion

The traditional treatment of RAS includes glucocorticoids and immunosuppressive therapy. These medications have been applied as topical pastes, mouthrinses, and intralesional injections and systemically by the oral route. Side effects such as burning sensation at the site of application, transient taste disturbance, intermittent headaches, and rarely patchy hyperpigmentation of the oral mucosa as a result of topical immunosuppressive treatment were reported [6, 7]. Recent reviews of the literature showed that topical treatment with natural herbal medicines seemed to benefit RAS patients by reducing ulcer size, shortening ulcer duration, and relieving pain without severe side effects, but the authors concluded that the evidence remains insufficient. Well-designed and high-quality randomized controlled trials are required for further exploration [4, 5]. A device proposed to treat RAS is laser therapy. A review of literature showed that, in the majority of the patients, immediate pain relief and accelerated ulcer healing were observed following irradiation with lasers [3], but in another recent paper the authors concluded that although laser therapy is better in relieving ulcer pain and shortening healing time than placebo group or medical treatment group, high-quality clinical studies with large sample size must be performed [1]. Other aids have been studied in the literature to promote healing of oral lesions including photodynamic therapy [8]. The FotoSan 630 has already been proposed in the literature for the treatment of periodontal and endodontic lesions but had not yet been tested on mouth ulcers [9, 10]. The use of photodynamic therapy for the oral soft tissues lesions is actually little studied, although it has been shown to be effective in other areas as in the periodontal treatment. The rapid improvement in symptoms reported by the patient and the increase of the aphthous lesion healing rate bode well for the use of this device in the treatment of this type of lesion, although clarification remains to be made of the healing methods that we found in this case. It is important to underline that the use of photodynamic therapy with FotoSan 630 could avoid the use of topical cortisone therapy, with its collateral events. Studies on FotoSan 630 have shown that its use is safe on oral tissues [11]. No other works in the literature report healing of aphthous lesion of this type following topical therapy or through the use of other principals. The purpose of this case report is to provide a starting point for the study of this phenomenon and more generally of the study of the application of FotoSan 630 device in the treatment of aphthous and oral soft tissue lesions.

## 4. Conclusion

Photodynamic therapy in the treatment of RAS could be considered safe and effective and could avoid the use of topical cortisone therapy, with its collateral events. Other studies are needed to explain this type of ulcer aphthous healing.
